# Specific recommendations to improve the design and conduct of clinical trials

**DOI:** 10.1186/s13063-023-07276-2

**Published:** 2023-04-10

**Authors:** Mark J. Kupersmith, Nathalie Jette

**Affiliations:** 1 Department of Neurology, Icahn School of Medicine at Mount Sinai, New York, NY USA; 2 Department of Ophthalmology, Icahn School of Medicine at Mount Sinai, New York, NY USA; 3 Department of Neurosurgery, Icahn School of Medicine at Mount Sinai, New York, NY USA; 4 Department of Population Health, Icahn School of Medicine at Mount Sinai, New York, NY USA

## Abstract

There are many reasons why the majority of clinical trials fail or have limited applicability to patient care. These include restrictive entry criteria, short duration studies, unrecognized adverse drug effects, and reporting of therapy assignment preferential to actual use. Frequently, experimental animal models are used sparingly and do not accurately simulate human disease. We suggest two approaches to improve the conduct, increase the success, and applicability of clinical trials. Studies can apply dosing of the investigational therapeutics and outcomes, determined from animal models that more closely simulate human disease. More extensive identification of known and potential risk factors and confounding issues, gleaned from recently organized “big data,” should be utilized to create models for trials. The risk factors in each model are then accounted for and managed during each study.

## Background

Why are not the results of drug clinical trials more effective? Except for certain infectious diseases treated with antimicrobial agents, medical therapies rarely cure illnesses. Rather, most therapies reduce risks, decrease the frequency of harmful consequences, and moderate the severity of future clinical deficits in specific patient populations. One example of a commonly used therapy, statins reduce low-density lipoprotein (LDL), which is associated with atherosclerosis plaque occurrence. But myocardial infarction is reduced only by approximately 30%, less if the drug is discontinued [1–4], The relationship between serum LDL levels and clinical benefit are not strong. Without addressing all of the other potential risk factors, we still do not know if LDL reduction, without other actions, is a true surrogate for clinical efficacy for statin use.

## Problems with current approaches to clinical trials

The often-modest clinical benefits of new FDA approved therapies may stem from the limitations of drug development, which includes fundamental problems in the design of many clinical trials [5–22]. Numerous critiques have detailed a variety of reasons why clinical trials frequently fail and the pitfalls for interpreting and applying the results [23, 24]. A partial list of these problems includes poor recruitment and retention, restrictive entry criteria that limit enrollment of patients with concomitant medical illnesses, children, older adults, and pregnant women; or non-participation by disenfranchised groups; brief study period and follow up; and failure to account for unrecognized adverse drug effects, some of which may be due to concomitant use of multiple non-study drugs. Other problems include using “intention to treat” as the principal analysis rather than the actual drug or intervention that occurred for each participant, or uniform dosing of study drug, which may not reflect actual blood and tissue levels [25]. Lastly, the selected endpoints may not be clinically meaningful. For example, with new cancer studies of people treated with immunotherapies, trials show a cellular immune response or a change in tumor burden, but the real goals are increasing survival and cures.

## Complexity of human disease

Current randomized trials are most often designed to show the effect of a specific therapy, drug, or procedure, compared with a placebo or one another, and less commonly two, therapy. A primary outcome is required, and numerous secondary outcomes are considered to measure the effect of the study intervention. Trials assume that for the duration of the study, participants will be otherwise stable except for the specific illness being investigated. The problem is that human diseases are often the results of numerous, often incompletely understood factors. We are complex living organisms, affected by an incalculable number of biological, behavioral, and environmental variables. Thus, it is improbable that a single therapy will completely control and certainly unlikely to cure most illnesses. Yet, investigators persist in this approach, choosing obtainable goals, but not necessarily proving a clinical benefit for study therapies. For example, most if not all medications approved for glaucoma are based on reducing elevated intraocular pressure. However, glaucomatous optic nerve damage and associated vision loss often occur despite lowering the elevated intraocular pressure. And we do not know how well the surrogate measurement of intraocular pressure is linked to the desired outcome of treatment (prevention of vision loss). In another example, it is yet to be determined whether drug induced activation of the immune system against cancer is strongly correlated with improved survival [26].

We suggest that the rigid tactics used in drug development accounts for some of the failures of drugs that appear to be successful in experimental animal disease models [27]. Additionally, promising drugs, which might be effective if combined with one or more additional interventions, are abandoned. Though the current approach has been effective for developing treatments for many disorders, it is clearly limiting.

Further, most disorders have multiple pathophysiological mechanisms that occur sequentially, simultaneously, or in other sequences. Altering or blocking one pathological process may not be adequate to result in clinical benefit. Recent trials in oncology demonstrate the benefit of combining chemotherapy and immunotherapy for reducing neoplasia pathology, but this does not always prolong survival [28], and, in most situations, cure rates remain low [29]. In another disorder that was virtually 100% life-ending, HIV antiretroviral therapy is effective, but HIV infection persists, so interventions to augment the immune response, such as the CD8 + T-cell response, are being tested [30].

Pharmaceutical and device companies want to have simple outcome measures and endpoints. The goal is to have short duration studies and secure regulatory agency approval and a marketed product. The US federal funding agency-supported trials are approved based on reviewers who demand simple unambiguous outcomes and brief duration trials. In general, few trials are designed to address and treat multiple factors, specifically targeted to individual participants (e.g., precision medicine) or addressing the features of models derived from prior studies or “big data” [31].

## Rethinking study design

Accounting for and managing identified risk factors is complicated and remains a major challenge in clinical practice. In the current environment, few healthcare providers have the time to adequately explore these issues, but there are efforts to address these complicating concerns to determine the effectiveness of therapies [32]. Providing the most complete phenotypes (determined by all features) for study stratification should be optimized. Of course, study participants deserve the best and safest management during every clinical trial, and this could be an added benefit to improve recruitment. Many trials utilize stratification to balance treatment groups for specific risk factors, but this is only a partial solution [33]. Instead of performing analyses such as proportional hazard on collected data, clinical trials should prospectively manage these factors. This would include assessing adherence of the prescribed management, in addition to the steps utilized to assess study drugs and safety. The results of such studies could provide the foundation for improved healthcare outside of clinical trials. The management of every risk factor does not necessarily require medications, as interventions such as lifestyle changes and behavioral counseling are beneficial for numerous diseases (e.g., obesity reduction for diabetes mellitus, systemic hypertension, and idiopathic intracranial hypertension). Providing management of many risk factors during a clinical trial may have an unintended consequence—an otherwise effective study therapy may not appear to be a robust treatment if each treatment group has additional effective interventions (e.g., the NIH sponsored Idiopathic Intracranial Hypertension Treatment Trial barely showed acetazolamide improved the primary outcome measure, a global index visual field loss, as the placebo group had managed weight reduction, a key treatment in the disorder [34]). Even if risk factor control might mask the effectiveness of a drug, if the drug is truly useful, it should be effective in participants who do not have the additional risk factors or in those who are more adherent to the self-management and recommendations for best medical care practices. Industry partners may be less responsive to this concept, as it could certainly drive up the costs of clinical trials.

Another problem in drug development is the use of experimental animal models that may not closely mirror the human disease to be targeted. Preclinical studies reasonably focus on safety, but drug development that bypasses use of the best animal models may lose valuable insight into the efficacy, the outcome measures, and dosing. Companies may accelerate this process to satisfy investors by rushing investigations of their study drugs into human clinical trials. Experimental animal models of disease rarely replicate human diseases, but they provide insight and identify multiple potential mechanisms in the pathophysiological process to possibly address (e.g., experimental autoimmune encephalomyelitis models for multiple sclerosis, laser vascular injury for ischemic optic neuropathy) [35]. Yet, results of human clinical trials and animal studies frequently differ, which may be due in part to differences between species, inadequate models, lack of consistent outcome measures, and inadequate sample size for the experimental model [36].

Recently, efforts by data/computer companies, healthcare systems, and other institutions have recognized the importance and power of using artificial intelligence and other methods to mine large patient databases and validate or develop new theories of risk factors, modifiable or not, for diseases. “Big” data can be used to create models, based on human data, which can be evaluated for significance in large databases such as IBM Market Watch or Medicare data. For example, a model can be used to determine the impact on developing a specific disorder over a defined time interval. Individuals with and without the features of the model, without the target illness at a starting time point, can be evaluated for the development and severity of the target disease in already collected global health data. Once the relevance of these human models is established, they can be applied to treatment trials. Machine and deep learning methods have been suggested as a way to identify potential clinical trial participants, augment stratification to reduce the number of patients needed, enhance recruitment, and streamline monitoring [37, 38]. To date, artificial intelligence-derived complex models of disease, using human and experimental paradigms, have been used sparingly to identify the multiple risk factors for clinical trials, and they have not been applied to develop the needed multiple approaches to treat illnesses. Using rheumatoid arthritis investigations as examples of this approach, studies show that that these investigations are not truly global in consideration of all potential factors. They tend to only take into account known features, thought to have potential for disease prevention [39] or to be associated with the disorder [40]. Thus, they do not seem to improve prediction of therapeutic response [41]. However, machine learning methods that utilize approaches such as the least absolute shrinkage and selection operator and random forests methods may identify new previously unrecognized predictors of disease severity and response to a therapy [42]. Clinicians and clinical trial experts in each specialty should contemplate using these “big data” to create expanded risk models that include more than the obvious risk factors. These models could be considered in clinical trial design and participants accordingly stratified at study enrollment. Treatment groups could include groups without potential risk factors and those with the risk factors, with further stratification depending on the degree of control of those factors.

## Conclusions

The proposed approaches must be and can be assessed before implemented in clinical trials. In non-medical fields such as finance, theoretical paradigms developed by academics have not always been successful in real world applications. The “science” of investing is replete with complex models that work in isolated situations but do not withstand the frequent shifting conditions in financial markets [43]. The chaos induced by the unpredictability of politics, regulations, wars, social changes, and weather defy accurate forecasting. Human health is similar—as health status is affected by stress, diet, and use of medications that can induce adverse effects. In contrast to financial market forecasting, comprehensive preclinical testing and the approach of testing the disease models in existing huge databases can overcome some of these issues and provide a form of replication. However, in the end, no database on human health is complete enough to absolutely account for every combination and permutation of human health. Table 1 lists the additional recommendations for improving future clinical trials.

**Table 1:** Recommendations to improve clinical trials

1. Develop experimental models that closely mirror the human disease for testing
2. Use of outcomes that reflect the animal model results and are clinically meaningful
a. Increased use of secondary outcomes when clinically meaningful
3. Dosing better tied to animal model outcome testing (in addition to safety)
a. Determine if drug levels in an accessible specimen are relevant to a response
4. Recognizing the complexity of human disease
a. Use existing databases to determine the risk factors that could be important in the study disease and create disease models
b. Manage other disease risk factors during the trial
c. Control for complications due to non-study medications
5. Combine therapies to address multiple pathophysiological mechanisms
